# Artificial intelligence for direct-to-physician reporting of ambulatory electrocardiography

**DOI:** 10.1038/s41591-025-03516-x

**Published:** 2025-02-10

**Authors:** L. S. Johnson, P. Zadrozniak, G. Jasina, A. Grotek-Cuprjak, J. G. Andrade, E. Svennberg, S. Z. Diederichsen, W. F. McIntyre, S. Stavrakis, J. Benezet-Mazuecos, P. Krisai, Z. Iakobishvili, A. Laish-Farkash, S. Bhavnani, E. Ljungström, J. Bacevicius, N. L. van Vreeswijk, M. Rienstra, R. Spittler, J. A. Marx, A. Oraii, A. Miracle Blanco, A. Lozano, I. Mustafina, S. Zafeiropoulos, R. Bennett, J. Bisson, D. Linz, Y. Kogan, E. Glazer, G. Marincheva, M. Rahkovich, E. Shaked, M. H. Ruwald, K. Haugan, J. Węcławski, G. Radoslovich, S. Jamal, A. Brandes, P. T. Matusik, M. Manninger, P. B. Meyre, S. Blum, A. Persson, A. Måneheim, P. Hammarlund, A. Fedorowski, T. Wodaje, C. Lewinter, V. Juknevicius, R. Jakaite, C. Shen, T. Glotzer, P. Platonov, G. Engström, A. P. Benz, J. S. Healey

**Affiliations:** 1 Department of Clinical Sciences, Malmö, Lund University, Lund, Sweden; 2 Population Health Research Institute, McMaster University, Hamilton, Ontario Canada; 3 Medicalgorithmics S.A., Warsaw, Poland; 4 Vancouver General Hospital, University of British Columbia, Vancouver, British Columbia Canada; 5 Karolinska Institutet, Stockholm, Sweden; 6 Department of Medicine Huddinge, Karolinska University Hospital, Stockholm, Sweden; 7 Department of Cardiology, Copenhagen University Hospital—Rigshospitalet, Copenhagen, Denmark; 8 Department of Medicine, McMaster University, Hamilton, Ontario Canada; 9 University of Oklahoma Health Sciences Center, Oklahoma City, OK USA; 10 Cardiology Department Hospital Universitario La Luz, Madrid, Spain; 11 Department of Cardiology and Cardiovascular Research Institute Basel, University Hospital Basel, University of Basel, Basel, Switzerland; 12 Department of Cardiology, Assuta Ashdod University Hospital, Ben-Gurion University of the Negev, Ashdod, Israel; 13 Department of Cardiology, Clalit Health Services, Tel Aviv Jaffa District, Israel; 14 Division of Cardiology, Scripps Clinic, San Diego, CA USA; 15 Arrhythmia Clinic, Skåne University Hospital, Lund, Sweden; 16 Clinic of Heart and Vessel Diseases, Institute of Clinical Medicine, Faculty of Medicine, Vilnius University, Vilnius, Lithuania; 17 Department of Cardiology, University of Groningen, University Medical Center Groningen, Groningen, the Netherlands; 18 Department of Cardiology, University Medical Center Mainz, Johannes Gutenberg-University Mainz, Mainz, Germany; 19 Department of Internal Diseases, Bashkir State Medical University, Ufa, Russia; 20 Feinstein Institutes for Medical Research at Northwell Health, Manhasset, NY USA; 21 Department of Cardiology, University Hospital of Zurich, Zürich, Switzerland; 22 Department of Cardiology, Centre hospitalier de l’Université de Montréal—Université de Montréal, Montréal, Quebec Canada; 23 Department of Cardiology, Cardiovascular Research Institute Maastricht (CARIM), Maastricht University Medical Centre, Maastricht, The Netherlands; 24 Faculty of Health and Medical Sciences, Department of Biomedical Sciences, University of Copenhagen, Copenhagen, Denmark; 25 Department of Cardiology, Gentofte Hospital, Hellerup, Denmark; 26 Department of Cardiology, Zealand University Hospital, Roskilde, Denmark; 27 Hackensack University Medical Center, Hackensack, NJ USA; 28 Hackensack Meridian School of Medicine, Nutley, NJ USA; 29 Department of Cardiology, Esbjerg Hospital—University Hospital of Southern Denmark, Esbjerg, Denmark; 30 Department of Regional Health Research, University of Southern Denmark, Esbjerg, Denmark; 31 Department of Electrocardiology, Institute of Cardiology, Faculty of Medicine, Jagiellonian University Medical College, Kraków, Poland; 32 St. John Paul II Hospital, Kraków, Poland; 33 Division of Cardiology, Department of Medicine, Medical University of Graz, Graz, Austria; 34 Department of Clinical Physiology, Skåne University Hospital, Malmö, Sweden; 35 Department of Cardiology, Helsingborg Hospital, Helsingborg, Sweden; 36 Department of Cardiology, Karolinska University Hospital, Stockholm, Sweden; 37 University of Glasgow, University of Glasgow, Institute of Wellbeing, Glasgow, UK; 38 Department of Clinical Sciences, Lund University, Lund, Sweden

**Keywords:** Arrhythmias, Diagnosis

## Abstract

Developments in ambulatory electrocardiogram (ECG) technology have led to vast amounts of ECG data that currently need to be interpreted by human technicians. Here we tested an artificial intelligence (AI) algorithm for direct-to-physician reporting of ambulatory ECGs. Beat-by-beat annotation of 14,606 individual ambulatory ECG recordings (mean duration = 14 ± 10 days) was performed by certified ECG technicians (*n* = 167) and an ensemble AI model, called DeepRhythmAI. To compare the performance of the AI model and the technicians, a random sample of 5,235 rhythm events identified by the AI model or by technicians, of which 2,236 events were identified as critical arrhythmias, was selected for annotation by one of 17 cardiologist consensus panels. The mean sensitivity of the AI model for the identification of critical arrhythmias was 98.6% (95% confidence interval (CI) = 97.7–99.4), as compared to 80.3% (95% CI = 77.3–83.3%) for the technicians. False-negative findings were observed in 3.2/1,000 patients for the AI model versus 44.3/1,000 patients for the technicians. Accordingly, the relative risk of a missed diagnosis was 14.1 (95% CI = 10.4–19.0) times higher for the technicians. However, a higher false-positive event rate was observed for the AI model (12 (interquartile range (IQR) = 6–74)/1,000 patient days) as compared to the technicians (5 (IQR = 2–153)/1,000 patient days). We conclude that the DeepRhythmAI model has excellent negative predictive value for critical arrhythmias, substantially reducing false-negative findings, but at a modest cost of increased false-positive findings. AI-only analysis to facilitate direct-to-physician reporting could potentially reduce costs and improve access to care and outcomes in patients who need ambulatory ECG monitoring.

## Abstract (web summary)

In a large-scale analysis of ambulatory electrocardiographic recordings, a deep learning algorithm had a substantially higher sensitivity for the detection of critical arrhythmias as compared to technicians, opening a path toward artificial intelligence-assisted direct-to-physician reporting of ambulatory electrocardiography results.

## Main

In recent years, there have been rapid developments in ambulatory electrocardiogram (ECG) technology that enable markedly increased use of ambulatory ECG monitoring. At the same time, the importance of detecting brief, infrequent arrhythmias, particularly atrial fibrillation (AF), has been recognized^1,2^. Longer ECG recording duration and frequency lead to higher detection rates of arrhythmia^3–6^, and extended ECG monitoring is recommended for patients with syncope^7,8^ and individuals in whom screening for AF to prevent new-onset or recurrent stroke could be beneficial^9^. The number of patients that may benefit from rhythm monitoring is also growing, particularly with evidence that short-duration subclinical AF^10^ may benefit from anticoagulation^1,11^. With the increasing availability of lower-cost devices, longer-term monitoring capabilities and the emergence of direct-to-consumer devices that provide irregular pulse notifications and record single-lead ECG intermittently, there has come a deluge of heart rhythm monitoring data that requires analysis^12,13^. Given the worldwide shortages of healthcare workers^14^, this increased workload may overburden human ECG technician resources, possibly reducing the quality of heart rhythm annotations^15–18^, leading to misdiagnosis, delayed treatment and adverse patient outcomes.

While it has widely been predicted that artificial intelligence (AI) will replace humans in some areas^19^, the nearest examples in healthcare are in mammography, where AI can replace a second physician reader for mammograms^20–23^, and in pathology, where AI tools improve pathologist accuracy and efficiency^24,25^. Implementation of an AI model that uses ECGs to alert physicians to high-risk hospitalized patients was recently shown to reduce mortality^26^, and several machine learning-based models that use ECG data to predict arrhythmia have been developed^27,28^. AI holds considerable promise for arrhythmia diagnostics as it can rapidly analyze a large amount of data at low cost, provide consistent annotations without risk of mental fatigue and provide results in near real time^29^. Previous studies indicate that AI algorithms can be trained to detect and accurately classify arrhythmias on resting ECG and ambulatory ECG recordings^30,31^, but no study has evaluated the role of AI in performing scanning and technical annotation of ambulatory ECG and providing results that can then be forwarded for physicians to review. Because AI-only reporting would mean that large amounts of ECG data would never be seen by a healthcare professional, such an AI model would need to have excellent negative predictive value for critical arrhythmias without generating unacceptable rates of false-positive annotations that would require physician review.

We designed the DeepRhythmAI for autonoMous Analysis of RhyThm INvestigatIon (DRAI MARTINI) study to test the DeepRhythmAI model for direct-to-physician reporting of ambulatory ECG data. The aim was to report on the performance of the DeepRhythmAI compared to technician analysis of ambulatory ECG data, including absolute rates of false-negative and false-positive detection for both the AI model and ECG technicians.

## Results

**Extended Data Fig. 1:**
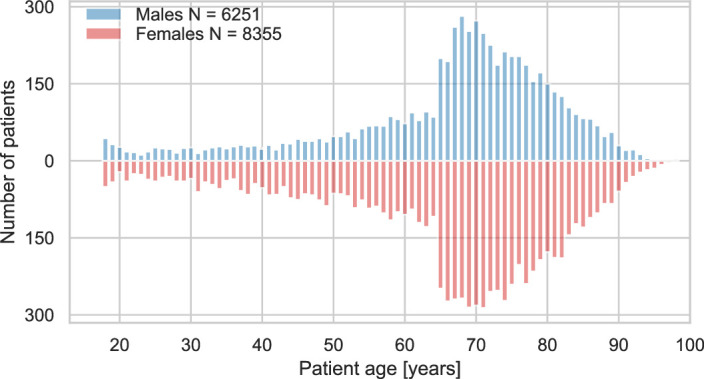
Age and sex distribution. Age and sex distribution of the patient sample included in the analyses.

The study population consisted of 14,606 patients (mean age = 65.5 ± 10 years, 42.8% males), who were monitored for a mean of 14 ± 10 days (Extended Data Fig. 1). Monitoring indications were provided through the device for 14,596 patients and are reported in Extended Data Table 1. The most common monitoring indications were palpitations, syncope, dizziness and examination for AF.

### Critical arrhythmias

**Fig. 1:**
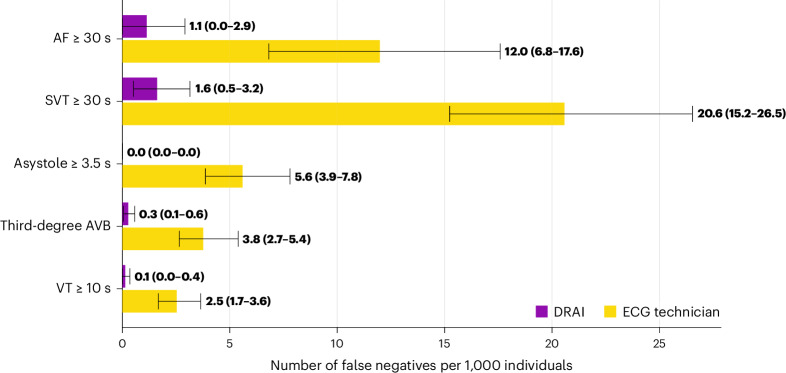
False-negative critical arrhythmias per 1,000 patients by AI and technician analysis. Error bars represent 95% CIs derived using bootstrapping. AVB, AV block.

**Fig. 2:**
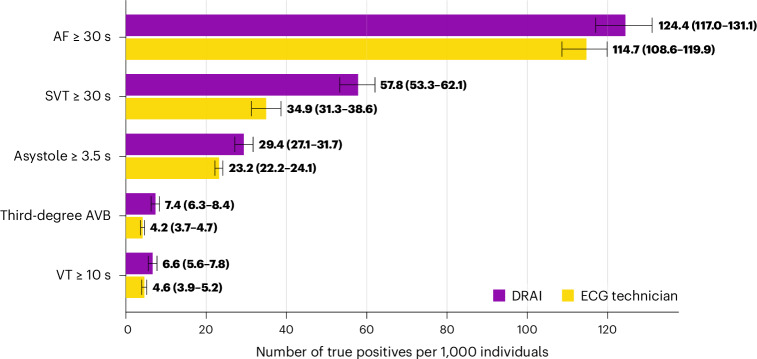
True-positive critical arrhythmias per 1,000 patients by AI and technician analysis. Error bars represent 95% CIs derived using bootstrapping.

**Extended Data Fig. 2:**
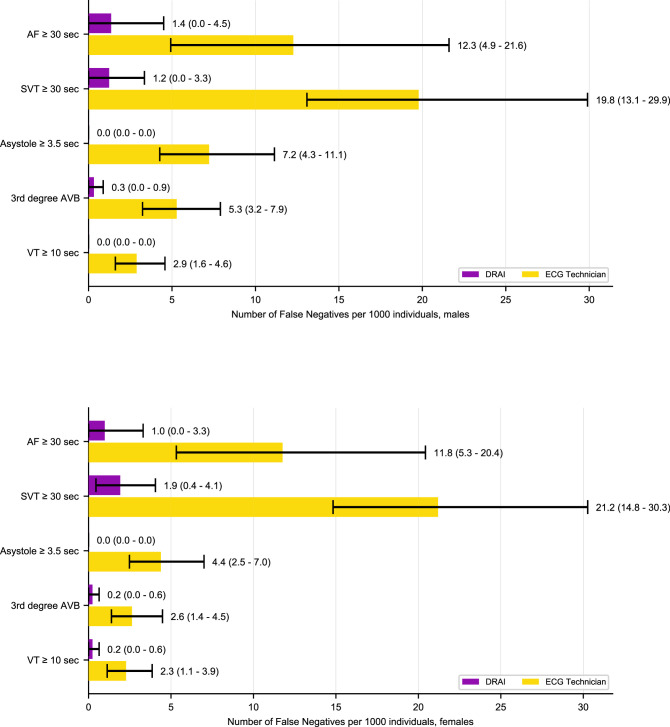
False-negative findings in males and females. Error bars denote 95% confidence intervals and were derived using bootstrapping.

**Extended Data Fig. 3:**
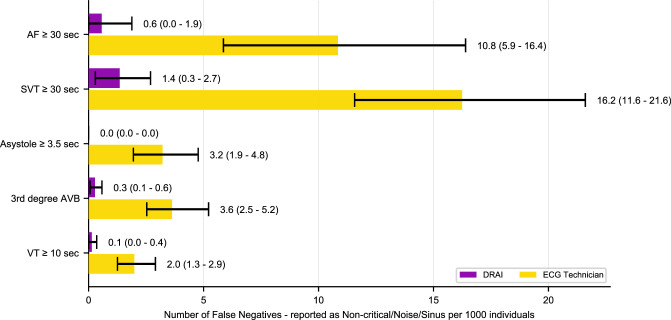
False-negative findings, excluding events reported as other critical arrhythmias. Error bars denote 95% confidence intervals and were derived using bootstrapping.

The AI model had superior sensitivity for the primary endpoint of false-negative findings (all instances of the arrhythmia missed for the full recording) of critical arrhythmia (98.6% (95% confidence interval (CI) = 97.7–99.4) versus 80.3% (95% CI = 77.3–83.3%); Table 1). This category includes ≥30 s of AF, ≥30 s of supraventricular tachycardia (SVT), sinus arrest/asystole events lasting ≥3.5 s, third-degree AV block of any duration and ≥10 s of ventricular tachycardia (VT) ≥ 120 beats per minute. The AI model analysis had 3.2 false negatives per 1,000 patients, compared to 44.3 per 1,000 for technicians (Fig. 1), resulting in a relative risk (RR) of a false-negative finding of critical arrhythmias of 14.1 (95% CI = 10.4–19.0) for technician analysis compared to DeepRhythmAI model analysis. Extended Data Table 2 reports these results for individual arrhythmias. The lower false-negative rate with the AI model was observed in both males and females (Extended Data Fig. 2). In a sensitivity analysis where misclassifications between critical arrhythmias were not considered AI or technician false negatives, we saw largely unchanged results—2.3 false-negative findings per 1,000 patients using the AI model and 39.4 per 1,000 patients for technicians (RR = 16.9 (95% CI = 12.0–23.9); Extended Data Fig. 3). This RR for false-negative findings over the full recording increased with increasing monitoring duration (RR = 7.8 (95% CI = 3.1–19.8) for 1–2 days of monitoring, RR = 9.1 (95% CI = 3.9–21.1) for 3–7 days of monitoring and RR = 17.9 (95% CI = 11.9–26.9) for ≥8 days of monitoring). Overall, the negative predictive value for critical arrhythmias was 99.9% (95% CI = 99.9–100%) for the AI model compared to 99.1% (95% CI = 98.9–99.2) for technicians, and the AI model had superior negative predictive values for all individual critical arrhythmia classes (Table 1). The AI model detection rates of true-positive VTs, SVTs, asystoles and third-degree AV blocks were substantially higher than the technicians, and the AI model detected numerically more AF events (Fig. 2). Episode durations for false-negative events are reported in the Extended Data Table 3.

**Table 1:** Performance of DeepRhythmAI and ECG technicians compared to the consensus panel of cardiologists for critical arrhythmias

	Accuracy (95% CI), %		True-positive rate/sensitivity, % (95% CI)		True-negative rate/specificity, % (95%CI)		PPV, % (95% CI)		NPV, % (95% CI)		F1 score, %	
	AI	Technician	AI	Technician	AI	Technician	AI	Technician	AI	Technician	AI	Technician
Overall average critical arrhythmias	98.1 (97.9–98.2)	98.4 (98.1–98.5)	**98.6 (97.7**–**99.4)**	80.3 (77.3–83.3)	98.1 (97.9–98.2)	**99.2 (99.0**–**99.3)**	71.3 (68.5–73.9)	**82.7 (79.4**–**85.6)**	**99.9 (99.9**–**100)**	99.1 (98.9–99.2)	82.7 (80.9–84.5)	81.5 (79.0–83.6)
VT ≥ 10 s	98.2 (98.1–98.3)	99.5 (99.4–99.6)	**98.0 (94.8**–**100)**	64.4 (54.9–73.8)	98.2 (98.1–98.3)	**99.8 (99.7**–**99.8)**	27.2 (22.8–32.3)	**67.7 (58.2**–**76.6)**	**99.98 (99.96**–**100)**	99.7 (99.6–99.8)	42.6 (37.1–48.6)	**66.0 (57.4**–**73.2)**
AF ≥ 30 s	97.2 (96.5–97.9)	97.4 (96.6–98.0)	**99.1 (97.7**–**100)**	90.5 (86.8–94.0)	96.9 (96.2–97.7)	**98.4 (97.8**–**98.9)**	82.3 (77.8–86.8)	88.9 (84.7–92.6)	**99.9 (99.7**–**100)**	98.6 (98.0–99.2)	90.0 (87.1–92.7)	89.7 (86.7–92.3)
SVT ≥ 30 s	97.4 (97.1–97.9)	96.1 (95.5–96.7)	**97.3 (94.9**–**99.1)**	62.9 (56.6–69.3)	97.4 (97.0–97.9)	98.1 (97.7–98.4)	70.6 (65.9–75.7)	65.8 (59.3–72.2)	**99.8 (99.7**–**99.9)**	97.8 (97.2–98.3)	**81.8 (78.3**–**75.2)**	64.3 (58.7–69.8)
Asystole ≥ 3.5 s	98.5 (98.2–98.7)	**99.2 (99.0**–**99.4)**	**100 (100**–**100)**	80.6 (75.0–86.0)	98.4 (98.2–98.6)	**99.8 (99.7**–**99.9)**	65.7 (60.5–70.4)	**91.2 (87.8**–**95.6)**	**100 (100**–**100)**	99.4 (99.2–99.6)	79.2 (75.4–82.6)	85.8 (82.1–89.5)
Third-degree AV block	99.3 (99.2–99.4)	99.5 (99.3–99.6)	**96.4 (92.5**–**99.2)**	52.6 (44.0–61.6)	99.3 (99.2–99.4)	**99.9 (99.8**–**99.9)**	51.2 (44.6–48.2)	**76.3 (67.1**–**85.4)**	**100 (99.9**–**100)**	99.6 (99.5–99.7)	66.9 (61.2–72.8)	62.2 (53.9–70.0)

The bold values denote nonoverlapping CIs between methods.

NPV, negative predictive value; PPV, positive predictive value.

**Fig. 3:**
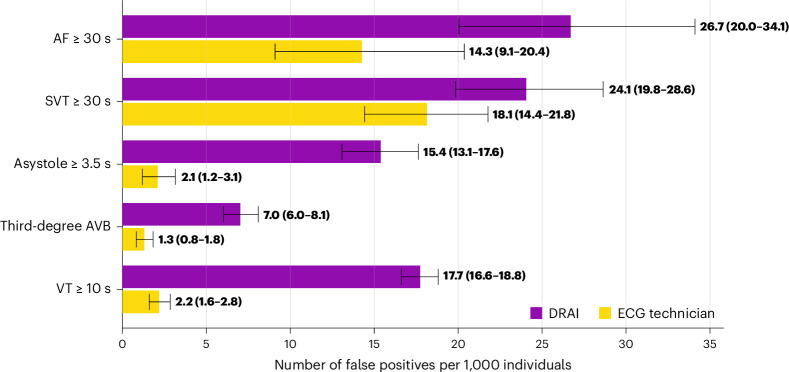
False-positive critical arrhythmias per 1,000 patients by AI and technician analysis. Error bars represent 95% CIs derived using bootstrapping.

**Fig. 4:**
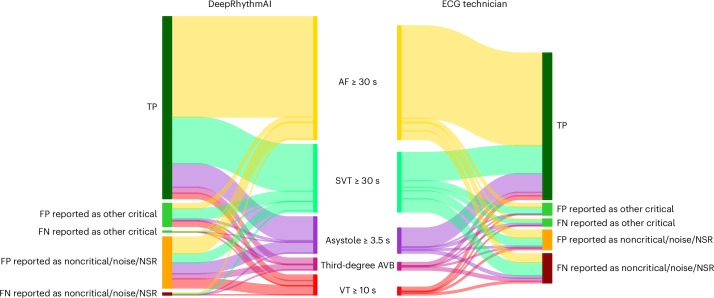
Diagnoses of patients with critical arrhythmias by DeepRhythmAI and ECG technicians. Sankey diagram showing arrhythmic event durations for critical arrhythmias as detected by each of the two methods. Cardiologist panel annotations are used to classify DeepRhythmAI and ECG technician annotations into TP, FP or FN. For FP and FN detections, we also report whether these were annotated by the cardiologist panels as another critical arrhythmia class or as a noncritical arrhythmia/noise or NSR. TP, true positives; FP, false positives; FN, false negatives; NSR, normal sinus rhythm.

**Extended Data Fig. 4:**
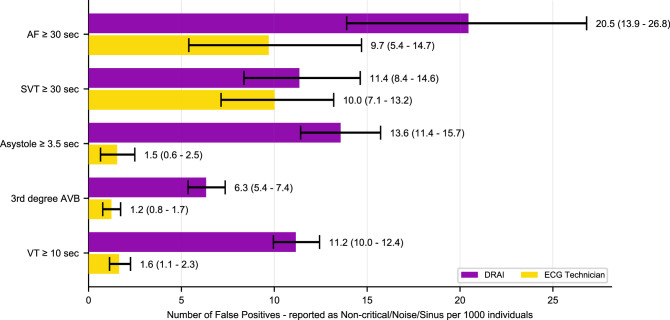
False-positive findings of critical arrhythmias. Error bars denote 95% confidence intervals and were derived using bootstrapping.

DeepRhythmAI model analysis resulted in more false-positive findings of asystoles, third-degree AV block and ≥10 s VT (Fig. 3). In sensitivity analyses when misclassifications between critical arrhythmias were not considered false positives, the total false-positive event rate over the full recordings was 6.3% for the AI model and 2.3% for technicians (Extended Data Fig. 4), corresponding to 12 (interquartile range (IQR) = 6–74) false-positive events per 1,000 patient days of recording for AI and 5 (IQR = 2–153) per 1,000 patient days of recording for technicians. Panel classifications of patients for whom strips were extracted are reported in Fig. 4. The duration of false-positive detections by the AI model and technicians is reported in Extended Data Table 3.

Full confusion matrix statistics for individual critical arrhythmias for both the AI model and technicians compared to panel annotations are reported in Table 1. DeepRhythmAI model analysis was superior in terms of sensitivity but had lower specificity for ≥10 s VT, asystole and third-degree AV block. The AI model analysis had similar positive predictive value to technicians for AF and sustained SVTs but lower positive predictive values for sustained VT, third-degree AV block and asystoles. The overall F1 score, which is the harmonized mean of positive predictive value and sensitivity, was similar for the AI model and technicians. However, the F1 scores for AI were superior for sustained SVT, and the F1 score for technicians was better for VT.

### Noncritical arrhythmias

Noncritical arrhythmias included premature atrial complexes and premature ventricular complexes, second-degree AV block, pauses of 2.0–3.5 s, VT episodes <10 s, idioventricular/accelerated idioventricular rhythms, SVT episodes ≤30 s and ectopic atrial rhythm. Results for these rhythm classes are reported in Table 2. The AI model had superior sensitivity for all noncritical arrhythmias and a superior F1 score for pauses and idioventricular/accelerated idioventricular rhythms but lower specificity for all noncritical arrhythmias except SVT episodes <30 s and ectopic atrial rhythms.

**Table 2:** Performance of DeepRhythmAI and ECG technicians compared to the consensus panel of cardiologists for noncritical arrhythmias

	Accuracy, % (95% CI)		Sensitivity, % (95% CI)		Specificity, % (95% CI)		PPV, % (95% CI)		NPV, % (95% CI)		F1 score, %	
	AI	Technician	AI	Technician	AI	Technician	AI	Technician	AI	Technician	AI	Technician
Second-degree AV block	92.3 (91.7–92.9)	**96.7 (95.7**–**97.4)**	**100 (100**–**100)**	38.6 (30.9–46.1)	91.9 (91.3–92.5)	**99.5 (99.3**–**99.7)**	41.9 (37.3–46.3)	**77.8 (68.9**–**87.0)**	**100 (100**–**100)**	97.1 (96.1–97.9)	59.1 (54.3–63.3)	51.6 (43.7–59.2)
2.0–3.5 s pauses	**95.0 (93.7**–**96.3)**	90.3 (87.4–92.5)	**97.8 (95.1**–**100.0)**	48.8 (40.1–57.7)	94.5 (93.0–96.0)	**97.8 (96.7**–**98.8)**	78.0 (71.9–84.3)	80.5 (70.4–89.3)	**99.5 (99.0**–**100.0)**	91.3 (88.1–93.6)	**86.8 (82.8**–**90.5)**	60.8 (52.0–68.0)
VT 3 beats, 10 s	81.9 (78.6–85.2)	84.7 (80.0–88.7)	**100.0 (100.0**–**100.0)**	58.3 (48.8–67.7)	75.8 (72.6–79.3)	**95.7 (93.6**–**97.8)**	58.5 (50.9–66.1)	**84.8 (76.8**–**92.4)**	**100.0 (100.0**–**100.0)**	84.7 (79.2–89.0)	73.8 (67.5–79.6)	69.1 (61.3–76.7)
AIVR	85.5 (82.6–88.4)	81.3 (77.2–84.8)	**100.0 (100.0**–**100.0)**	52.5 (42.4–62.0)	81.1 (78.1–84.3)	**90.1 (87.5**–**92.4)**	61.6 (53.8–69.2)	61.6 (50.6–71.1)	**100.0 (100.0**–**100.0)**	86.2 (81.5–90.2)	**76.2 (70.0**–**81.8)**	56.7 (47.3–64.6)
IVR	93.0 (92.0–94.1)	92.8 (90.4–94.6)	**100.0 (100.0**–**100.0)**	29.7 (22.1–38.2)	92.4 (91.4–93.5)	**98.9 (98.4**–**99.4)**	54.0 (47.5–61.1)	**72.9 (60.0**–**85.7)**	**100.0 (100.0**–**100.0)**	93.6 (91.0–95.4)	**70.1 (64.4**–**75.9)**	42.2 (32.9–51.1)
SVT 3 beats, 30s	79.1 (73.1–84.6)	76.9 (71.0–82.3)	**100.0 (100.0**–**100.0)**	90.3 (84.2–96.0)	55.8 (49.6–62.9)	62.9 (56.4–70.4)	71.6 (63.4–79.1)	71.8 (63.5–79.4)	**100.0 (100.0**–**100.0)**	86.2 (77.7–94.1)	83.5 (77.6–88.4)	80.0 (73.9–85.1)
EAR ≥ 3 beats	**83.0 (78.7**–**87.6)**	64.8 (59.4–70.2)	**99.1 (96.8**–**100.0)**	56.6 (45.9–67.4)	70.1 (65.0–75.9)	68.9 (64.7–73.2)	**72.7 (65.7**–**79.7)**	48.0 (38.0–58.0)	**99.0 (96.5**–**100.0)**	75.8 (68.7–82.2)	**83.8 (78.9**–**88.7)**	51.9 (43.0–60.5)

The bold values denote nonoverlapping CIs between methods.

AIVR, accelerated idioventricular rhythm; IVR, idioventricular rhythm; EAR, ectopic atrial rhythm.

## Discussion

This large, carefully adjudicated analysis demonstrates that the DeepRhythmAI model could safely replace technician interpretation of ambulatory ECG recordings, with an impressive sensitivity for critical arrhythmias and a modest increase of false-positive detections. The DeepRhythmAI model had a negative predictive value for critical arrhythmias that exceeded 99.9% and, compared to technicians, resulted in 17 times fewer patients with a missed diagnosis of a critical arrhythmia. This was at a cost of 2.4 times more false-positive detections, which for critical arrhythmias occurred once every 6 recordings for AI and once every 14 recordings for technicians. Considering that the DeepRhythmAI model performance exceeds the benchmarks of 99% negative predictive value and 70% positive predictive value that guidelines have recommended for accepting a single high-sensitive troponin to rule out major adverse cardiovascular events^32–36^, we consider DeepRhythmAI model-only analysis to be safe for the analysis of ambulatory ECG data.

The current study differs fundamentally from previous studies of AI for arrhythmia classification in that we evaluate the use of AI as the only reader for the majority of the health data, with physician confirmation only of AI model-selected episodes. This may be necessary for the management of the rising volume of ECG that will need to be accurately adjudicated without missing critical events. The sample size in terms of annotated strips in this study is 6–16 times larger than previous studies^30,31^, and the patient population negative predictive value, absolute false-positive and false-negative rates for AI-only analysis have never been reported before. These data are necessary to determine whether an AI can safely be used for direct-to-physician reporting and have not been shown in previous studies evaluating AI for arrhythmia diagnostics. Direct-to-physician reporting of ambulatory ECG results could unburden strained healthcare environments and result in an appropriate expansion of access, which should result in more equitable access to testing and subsequent care. We used a large, unselected clinical patient population to estimate how the use of the DeepRhythmAI model analysis instead of ECG technician analysis would affect the accurate detection and false-positive rates, using the beat-to-beat classification of a large and representative sample of arrhythmic events. Due to our sampling strategy, the measures of sensitivity that we report are not directly comparable to the sensitivity reported in selected rhythm strips in previous studies. We report as false negatives only patients in whom a diagnosis was missed for the full duration of the recording (that is, 14 ± 10 days of monitoring), arguably a more relevant evaluation metric. With this in mind, the AI model we evaluated had better sensitivity for all critical arrhythmias that were evaluated in both this study and a study assessing a deep neural network architecture for rhythm classification of single-lead ECGs^31^, a study evaluating a convolutional neural network for rhythm classification of 12-lead ECGs^30^ and a study comparing a deep neural network with physician over-reading of the full ECG to an electrophysiologist review of a traditional Holter system^37^. While the technician sensitivity in this study is low, this finding is in line with previous studies that show a low average accuracy in ECG interpretation for technicians^38^.

The large difference in false-negative findings using the DeepRhythmAI model and technician analysis could be dependent on factors related to algorithms and factors related to causes of human error. The higher rate of technician false negatives is likely in part to be due to limitations of features-based algorithms compared to AI models, but because technician work also includes scanning the ECG manually and assessing heart rate trends, there could also be effects of time pressure, information overload^15,17^ and other factors related to limits in human perception and memory^16,18^, which do not affect AI models. Thus, with increasing data volume that will require analysis, the AI model increasingly outperforms technician interpretation, giving consistent annotations not subject to fatigue. Rhythm analysis by technicians depends on correctly identifying and retaining in memory a large number of visual features; for example, a single capture beat in a wide complex tachycardia is pathognomonic for VT, but the human working memory has a fixed upper limit, and high information loads, such as in the analysis of ambulatory ECG recordings, can lead to reduced accuracy decision quality^15,16^.

Some limitations in study design should be considered. First of all, the technicians, but not the physician panels or the AI model, had access to clinical information such as monitoring indication, age and sex, which may have introduced a bias in favor of the technicians. At the same time, while the technicians were performing their analysis during paid clinical work hours, the cardiologist panels were performing their analysis as part of a research protocol, and therefore the panel annotations do not exactly represent a clinical workflow. Panel cardiologists may have been either more or less careful than they would have been with clinical patients, which could have introduced misclassification bias. We have not differentiated between second-degree AV block types 1 and 2, and we do not report subgroups by monitoring indication. Because monitoring indications were entered through the device, the absence of a reported indication should not be interpreted as a lack of that indication. The false-negative events in the study were patients in whom all episodes of arrhythmia were missed for the entire recording duration by one method, but at least one was detected by the other. While we consider this to be a robust method for false-negative estimation, it is possible that there are additional arrhythmic events that were undetected by both the AI model and technicians. If any arrhythmias were missed by both methods, this would imply a lower sensitivity and negative predictive value for both technicians and the AI model but not affect the results showing a superior sensitivity and negative predictive value for the AI model compared to technicians. It is also important to point out that, while the technicians were aided by a Food and Drug Administration-approved algorithm and also performed a manual review and reannotation of the data, their use of a different algorithm may have yielded different results. The underlying ECG data were recorded by a device providing leads II and III. However, the use of devices with nonstandard lead configurations and single-lead recording is becoming more prevalent. The results cannot be generalized to other AI algorithms, and the DeepRhythmAI model may have different performances on other signals, although, in view of the accuracy that the DeepRhythmAI model demonstrated in this study, the model could be tested on other ECG recording signals in the future. Finally, while we used an unselected patient population and extracted a large representative sample of relevant arrhythmic episodes for evaluation, some evaluation metrics that we report, such as the negative predictive value, are dependent on the population prevalence of arrhythmia, which may differ between different populations and may change over time.

Direct-to-physician reporting of leads II and III ambulatory ECG recordings using the DeepRhythmAI model would result in 17 times fewer missed diagnoses of critical arrhythmias than usual care with technician annotation and has a negative predictive value exceeding 99.9%. This would be at a cost of seven extra false-positive findings per 1,000 patient days of recording. AI analysis may substantially reduce labor costs and could potentially report results in near real time.

## Methods

### Data source

The source population for this study is an unselected patient population of 14,606 individuals, consisting of a random sample of patients who had been monitored in the United States for clinical indications between 2016 and 2019. Recording durations varied from 1 to 31 days. The dataset consisted of 211,010 days of ambulatory monitoring collected in these patients using PocketECG (Medicalgorithmics). PocketECG is a full-disclosure ECG device with limb lead configuration (leads II and III) and a sampling rate of 300 samples per second. The device can record and transmit ECG signals for up to 31 days. The patients were referred by 1,079 different physicians from 166 clinics, and the recordings were analyzed in clinical practice at an independent diagnostic testing facility by one of 167 certified ECG technicians working with a features-based algorithm using adaptive beat morphology template generation and comparison so that each QRS complex in the recording was annotated beat-to-beat by the ECG technician. ECG technician work was extensive and included a review of the whole ECG recording and verification of all events detected by the algorithm, including pauses and asystoles, all bradycardia events, all missed heartbeats or second- and third-degree AV blocks, all ventricular and supraventricular arrhythmias and all episodes detected as AF. In this process, artifacts and electrode dysfunction were re-annotated. The technicians also inspected all regions of the recording marked as having a ‘patient-triggered symptom’ flag and reviewed the recording at the time of the fastest, slowest and average minutely heart rate. They were aided in this process by software that allowed them to manually inspect heart rate trends for irregularities, filter beats by heart rate and group beats into morphologies. At the end of the review, episodes were selected for inclusion in a report to physicians.

Before inclusion in the study, all data were anonymized, and the Ethics Review Board of Sweden has therefore waived the need for approval (decision 2019-03227). As such, the Ethics Review Board did not consider that informed consent was necessary.

### DeepRhythmAI

**Extended Data Fig. 5:**
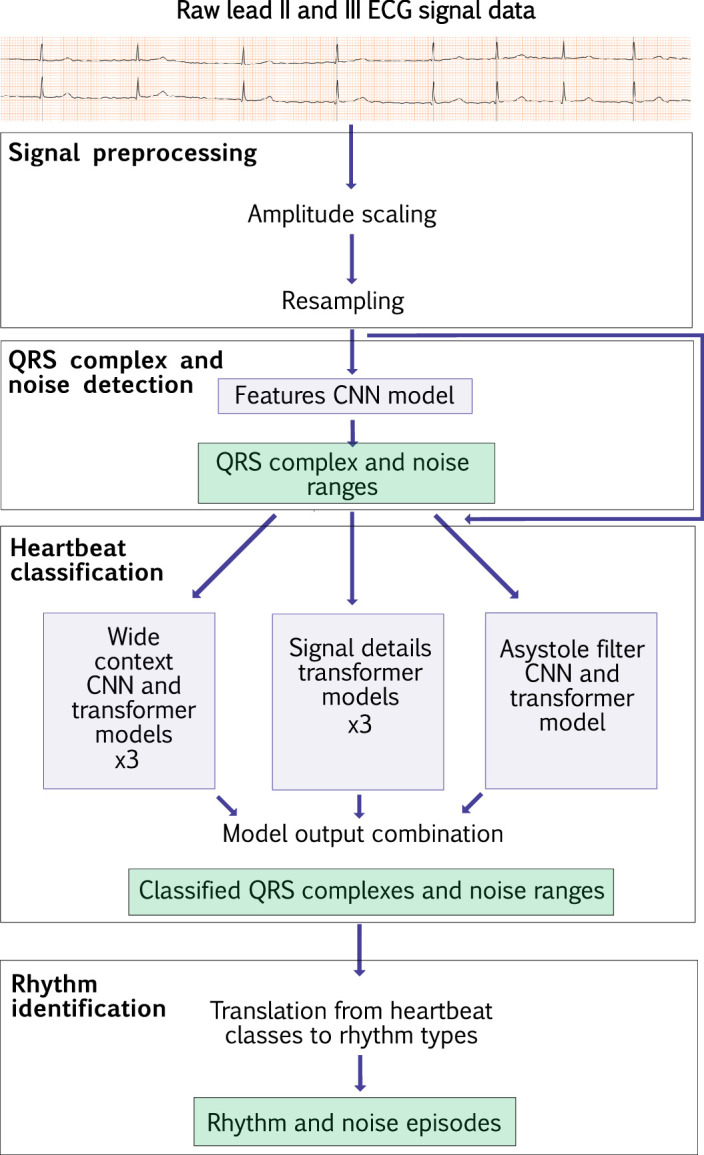
Schematic overview of the DeepRhythmAI model. The raw ECG signal (in timestamp + mV format) is pre-processed and fed to a single CNN classifier model that identifies the QRS complexes and segments of noisy (non-diagnostic) signals in the raw ECG data. The network components downstream to this module are fed both raw signal and the QRS/noise module output. This combined signal and QRS/noise data are processed by ensemble of a total of 7 models with both wide context (HR trend and morphology of beats) and narrow context (signal details). The wide context module is an ensemble of three custom deep neural network models with both CNN and transformer layers. The narrow context module is an ensemble of three transformer models all based on Vision Transformer ideas but with custom adaptations to 1D multichannel ECG signal. The output from these models is then combined with a wide context asystole filter that has the same architecture as wide context models but with hyperparameters tuned for asystole detection. The asystole filter overrides and replaces the other probabilities when asystoles are detected; otherwise, the output probabilities are averaged.

The DeepRhythmAI model (v3.1; Medicalgorithmics) is a proprietary mixed network ensemble for rhythm classification. The network performs QRS and noise detection, beat classification and rhythm identification using several algorithms based on convolutional neural networks and transformer architecture with custom-built components^39–42^. The main network components for QRS detection and rhythm classification have been pretrained on 1,716,141 5-min-long ECG strips and fine-tuned on 60,549 ≤30 s ECG strips. These were extracted from 69,706 anonymized clinical long-term recordings. Algorithm internal validation was performed using 15,188 ≤30 s strips from 12,330 additional separate patient recordings. A high-level flowchart of the algorithm is presented in the Extended Data Fig. 5. The preprocessing involves selecting desired ECG channels from input data, scaling the signal amplitude according to the input analog–digital conversion values and resampling to a frequency of 300 Hz. A deep learning model predicts the probability of QRS complex presence and signal readability, extracts signal features and predicts the probability of QRS complex presence and readable signal^39^. This output, together with the preprocessed signal, is passed to an ensemble combined from models of two structures. The first is intended for the analysis of information from a wide context and has a hybrid architecture of the convolutional neural network and transformer encoder layers^40^. The second is a pure-transformer implementation based on Vision Transformer^41^, allowing for a superior interpretation of signal within a relatively narrow window. Additionally, a specialized classifier was developed for the detection of asystole events.

The QRS complex detector uses custom residual modules inspired by MobileNetV2.^42^ Each module consists of the following three one-dimensional convolutional layers: a pointwise convolution to expand feature dimension; a convolutional layer with a kernel length of 3 and variable dilation rates; a pointwise convolution to reduce feature dimensions to their original size. The dilation rate doubles in each residual module during the first half of the model and then progressively decreases to a rate of 1 at the output layer. A final linear layer converts the output features into probabilities of QRS complex presence and signal readability for each sample. Thresholding and morphological operations are subsequently applied to extract QRS positions and identify nondiagnostic ranges. The wide-context architecture comprises a series of submodules. Initially, features are extracted from heart rate trends, calculated based on QRS detections, using the same architecture as the QRS detector (excluding the final linear layer). Another submodule extracts features for each sample of the preprocessed ECG signal using residual modules from the QRS detector but with a fixed dilation rate progression. The signal is downsampled using strided convolutional layers. Subsequently, windows of downsampled features are extracted, and two-dimensional strided convolutional layers are applied, resulting in features for each beat. The resulting features are processed using transformer encoder layers, augmented by an additional convolutional layer inserted between the linear layers in the fully connected blocks. Finally, the features are converted to logits for each QRS complex class using two pointwise convolutional layers.

The signal-detail architecture is based on transformer encoder layers that process ECG signals split into patches. A linear layer embeds each patch. The transformer layers process the embedded patches, and logits for each QRS complex class are calculated using a linear layer. Only the patches containing QRS complexes are selected for predictions. The asystole filter module shares the same architecture as the wide-context model but is trained with hyperparameters and a dataset tailored to the asystole detection task.

We used the same dataset for training the QRS complex and noise detector and the main components of the heartbeat classification ensemble (three wide-context models and three signal-detail models). Data augmentation techniques tailored to each of these tasks, like noise artifact generation or synthesis of heartbeats with rare features, were used to enhance training dataset diversity and mitigate overfitting. In addition to that, a classifier specializing in the interpretation of asystole events was developed by feeding to a single model with wide-context analysis architecture a carefully selected 11,670 strips with asystole or sinus arrest and 20,292 strips with noise or electrode dysfunction. The training process of this model encompassed methods from supervised and self-supervised learning domains. The ensemble model output is averaged or replaced by the asystole filter model output (for heartbeats with RR interval greater than the sinus arrest threshold of 2 s) to provide the probabilities of QRS complex classes. Finally, the heartbeat types that are the final output of the DeepRhythmAI model are translated to heart rhythm types. Optimization was performed using the AdamW algorithm. Models were internally evaluated by measuring the root mean squared error metric based on sensitivity, precision and F1 score calculated from predictions and ground truth of internal validation/test strips, following the methodology provided by the International Electrotechnical Commission 60601-2-47 standard^43^.

The ECG recordings used in this study had never been presented to the DeepRhythmAI model or any AI model from which the DeepRhythmAI model was derived, but as part of the study protocol, we analyzed the entire raw ECG signal data from these same recordings using the DeepRhythmAI model to provide detection and beat-to-beat classification of all heartbeats.

### Definition of critical and noncritical arrhythmias

#### Selection of representative arrhythmic episodes

**Extended Data Fig. 6:**
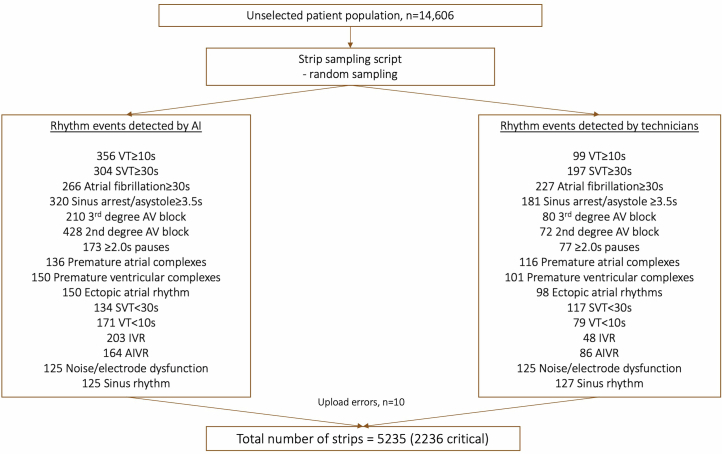
Description of the strip selection process. VT, ventricular tachycardia; AF, atrial fibrillation; SVT, supraventricular tachycardia; AIVR, accelerated idioventricular rhythm; IVR, idioventricular rhythm; EAR, ectopic atrial rhythm.

Our strip selection method was designed to not introduce any bias toward using ECG signals with less baseline noise or arrhythmic events presenting with typical ECG diagnoses. We did this by automation; fully random individual recordings were searched by an algorithm for the presence of arrhythmic events of each rhythm class, and 34-s strips containing arrhythmia events according to either the AI model annotations, the ECG technician annotations or both were selected, at a maximum of one per method and arrhythmia class per patient. The automated selection script ran until a total of 500 strips each had been selected for each of the critical arrhythmias and 250 strips each had been selected for the noncritical rhythm classes, or all recordings had been searched and no more arrhythmias were found. The number of individual recordings that had to be searched to yield the strips for each rhythm class was considered the source population size for that class. The strip selection is described in Extended Data Fig. 6. In addition to the critical and noncritical rhythm classes, we included sinus rhythm, sinus bradycardia and unreadable signals due to noise or electrode dysfunction to evaluate the AI model performance for these signals and to ensure that the physician annotators would be provided with a differentiated sample in which they did not know which strips would contain critical arrhythmias. In total, we selected 5,245 strips, of which 2,240 were critical arrhythmias, and after errors in uploading ten of these to the annotation platform, we had 5,235 strips, of which 2,236 were critical arrhythmias.

#### Consensus panel annotations

All 34-s strips were annotated beat-to-beat by 17 panels consisting of three expert annotators each—≥2 board-certified cardiologists and additionally including board-certified clinical physiologists (*n* = 2) or final-year cardiology residents. The physicians on the panels performed the annotation independently of AI and technician annotations and were blinded to the strip selection criteria. Strips were randomly distributed among panels and presented in random order and were annotated using a custom-built software platform in which QRS complex tags, without beat type classifications, as detected by the AI model, were present. We used DeepRhythmAI model-detected QRS complexes for strips detected by both the AI model and the technicians to minimize bias; technicians in clinical practice may not have bothered to correct QRS tags for all instances of arrhythmia, and differential methodology for strips could have resulted in unblinding. The QRS tags were highly concordant. For QRS complexes that resulted in technician false negatives, there was a 98% overlap between the AI model and fixed features algorithm QRS positions. Physician annotators were asked to identify the beat type for each QRS complex according to an annotation manual (Supplemental Note), correct any mistaken QRS position placements, add any missed QRS complexes and mark areas that were unreadable due to poor signal or electrode dysfunction. Each physician annotated the entire strip beat by beat, and all discrepancies on the beat level were resolved by panel consensus. The resulting gold-standard annotations were compared to the beat-to-beat annotations of the AI model and technicians according to prespecified acceptance criteria, where we considered arrhythmic events to be concordant with the panel annotation in case of ≥80% overlap in beat type and duration with the panel annotation for all sustained tachyarrhythmias and 90% overlap in duration for asystole events and pauses. For second- or third-degree AV block, we considered the presence of any such event within the strip to be a concordant annotation, and for ECG technicians, we also considered annotation of an unspecified ‘missed beat’ to be a concordant annotation for second-degree AV block. Single ectopic atrial and ventricular beats were considered concordant within ±45 samples (150 ms). Noise annotations were considered concordant if within 80% of the panel annotation as regards duration. Minor discrepancies between the AI/technician annotations and consensus panel annotations, on the beat-to-beat level, were thus allowed, for example, low numbers of supraventricular beats or beats with unknown beat types within AF episodes.

#### Statistics

The primary analysis compares the frequency of false-negative, true-positive and false-positive critical arrhythmias per 1,000 individual patients over the full duration of the recordings for technicians and the AI model, along with full confusion matrix statistics for the AI model and technician performance compared to panel annotations. As a result of the sampling strategy, false negatives were only reported in patients in whom all instances of an arrhythmia type were missed for the entire duration of the recording. True-positive events were defined as episodes detected by the AI model or technician, with correct annotations according to the independent gold-standard consensus panel annotation. Descriptive statistics are reported as mean ± s.d. CIs were derived using bootstrapping with 1,000 replications. Definitions for the confusion matrix statistics are reported in the Extended Data Table 4. We also performed subanalyses where misclassifications of critical arrhythmias were not considered false-negative or false-positive events because these events would have been reported to physicians. In these analyses also, we did not consider second-degree AV block to be a false-positive finding. For the analyses of total false-positive and false-negative findings of critical arrhythmias, the prevalence of all arrhythmias was weighted to the full population size according to the proportion of the population queried. Nonoverlapping CIs were considered evidence of the superiority of one method over the other. All analyses were performed in Python, except for the calculations of RR, which were done in Stata version 17.0 for Mac, using two-sided Fisher’s exact *P* values. Analyses were performed by L.S.J. and G.J., with involvement from the steering group, according to prespecified plans. The study steering group (L.S.J., J.S.H., A.P.B. and A.M.) met regularly throughout the conduct of the study without the presence of Medicalgorithmics employees.

### Reporting summary

Further information on research design is available in the Nature Portfolio Reporting Summary linked to this article.

## Online content

Any methods, additional references, Nature Portfolio reporting summaries, source data, extended data, supplementary information, acknowledgements, peer review information; details of author contributions and competing interests; and statements of data and code availability are available at 10.1038/s41591-025-03516-x.

## Supplementary information

Supplementary Information Supplemental Note.

Reporting Summary

## Figures and Tables

**Extended Data Table 1:**
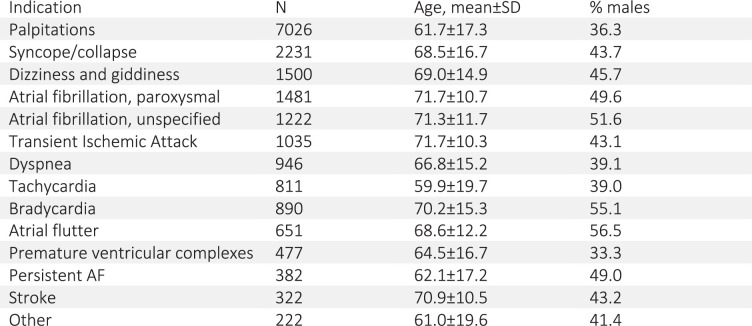
Monitoring indications reported on device

Several answers per patient possible.

**Extended Data Table 2:**
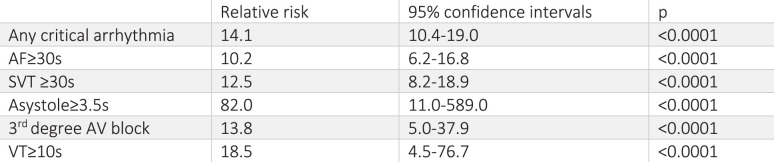
Relative risks of false-negative findings for technicians compared to artificial intelligence

AI analysis resulted in no false negatives for asystoles ≥3.5 seconds, results are calculated with the addition of 1 false negative for AI in this category. Two-sided *P* values were derived using Fischer’s exact test. VT, ventricular tachycardia; AF, atrial fibrillation; SVT, supraventricular tachycardia.

**Extended Data Table 3:**
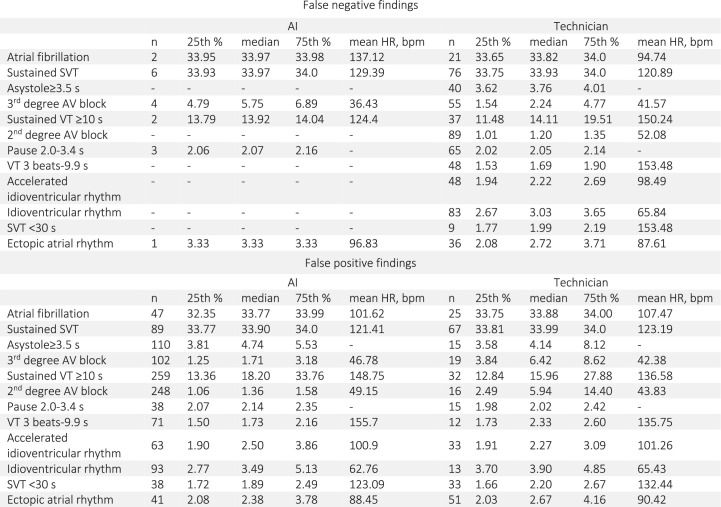
Duration and heart rate of false-negative and false-positive findings by either method

All durations are presented as median (interquartile range) in seconds, and all heart rates are presented as means. SVT, supraventricular tachycardia; VT, ventricular tachycardia; HR, heart rate.

**Extended Data Table 4:**
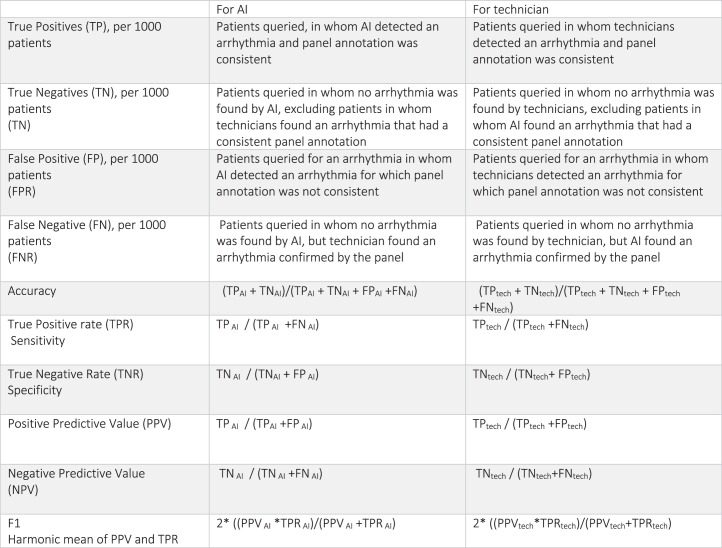
Confusion matrix definitions

Describes how the confusion matrix statistics are calculated from the annotated episodes.

## Data Availability

The data that supports the findings of this study are derived from patient ECGs and are not publicly available due to privacy concerns but will be made available after a request for access to the corresponding author for the purpose of reviewing the study results and at the cost of a data preparation fee. No requests that include a commercial interest will be approved. Data are located in controlled access data storage at Medicalgorithmics. A response to a request to access the data can be expected within 2 months.

## Code availability

The code for the DeepRhythmAI model is not available due to its proprietary nature. The code used for statistical analyses will be made available upon request to the corresponding author for the purpose of reviewing the results in the paper, and a response can be expected within 2 months.
