# Supplementary material for: Artificial intelligence for direct-to-physician reporting of ambulatory electrocardiography
Source: Nat Med. 2025 Feb 10;31(3):925–31. doi: 10.1038/s41591-025-03516-x (PMC11922735; doi:10.1038/s41591-025-03516-x)
Supplement: Supplementary file 1 — Supplemental Note. [file 41591_2025_3516_MOESM1_ESM.pdf]

nature medicine

Supplementary information https://doi.org/10.1038/s41591-025-03516-x

Artificial intelligence for direct-to-physician reporting of ambulatory electrocardiography

In the format provided by the authors and unedited

Supplemental data and information “Artificial Intelligence for Direct-To-Physician Reporting of Ambulatory Electrocardiography”

Supplemental Note 1

DRAI MARTINI Annotation manual

Basic information

These ECGs are registered using the PocketECG device, which is a full disclosure MCT device with limb leads with ECG leads II (top tracing) and III (bottom tracing). Deep Rhythm AI (DRAI) is built to detect QRS complexes, and then assign beat type labels to them. In the annotation tool, the Techbot teacher, all annotation is therefore has to be done beat by beat. QRS detection will be performed by DRAI, but you will need to provide labels to the beats that identify their type. After that, the system will assign rhythm classes. For example, five PVCs in a row at a rate ≥120 bpm will be classed as a non-sustained VT, and in a similar way the system will identify bigeminy and trigeminy episodes. DRAI can be wrong in four different ways – misclassifying a beat that exists, classifying noise as beats, missing real beats or inaccurately detecting a QRS that isn't there. Techbot teacher will provide a tag for each R wave. As expert annotator we are asking you to:

1. Provide the correct beat type label for every beat.
2. Remove QRS tags for noise that was erroneously identified as a QRS complex.
3. Add QRS tags for missed beats, or adjust the R-tagging.
4. Mark all instances of non-diagnostic signal or electrode disconnection

QRS position

We don't expect that many cases will need QRS correction, but if you should need to please mark the beat position near the middle point of the QRS complex. Avoiding conflict cases In your annotation teams you will also need to resolve all conflict cases, i.e., strips in which ≥1 beat is not uniformly labeled by all annotators. In order to minimise these it is important to be a bit careful with onset and offset of arrhythmias. For example, an AF episode that starts with two premature atrial complexes should be annotated with two premature atrial complexes at the start. A fusion beat within a VT episode should be marked as a fusion beat.

Assigning beat labels

There are two types of beat labels that should be considered, in order to accurately describe arrhythmic events - first and second level classes. First level beat labels describe the origin of the beat, while second order labels describe other characteristics related to the arrhythmia type, and includes aberration, AV-block, pre-exitation, pacing and sinus arrest. All beats need one first level class, but the second level is used only when needed. There are also area annotations - these are used when QRS complexes can't be discerned during either noise, electrode dysfunction or ventricular fibrillation.

First level labels

- **N** – sinus beat. These should have positive p-waves in lead II (top) and be used in normofrequent (50-100 bpm) sinus rhythm, sinus bradycardia, and sinus tachycardia, but also for sinus beats following pauses or asystole events.
- **AF** – atrial fibrillation/flutter beat. Irregularly irregular rhythm without p-waves or an isoelectric baseline, or a rhythm with flutter waves. AF annotation should begin at the first AF beat and end at the last. If clear sinus rhythm episodes are interpolated within AF episodes these should be marked as sinus beats. Note that any rhythm that has these characteristics should be marked as AF, even if shorter than 30 seconds. The system will subdivide episodes of AF into AF≥30 seconds and micro-AF (<30 seconds of typical AF).
- **S** –supraventricular ectopic beat, including premature atrial complexes, supraventricular tachycardias, other ectopic atrial rhythms such as AVNRT and AVRT, low atrial rhythms (with the P-wave before the QRS complex) escape rhythms, competing atrial foci and supraventricular beats that follow pauses. Mark all such beats as S, and these will be sorted by the system into singles, couplets, SVT runs and bi/trigeminy episodes according to pattern and rate of occurrence. Note: Mark as S episodes of AET with blocked conductions, ex. 2:1.
- **J** – junctional beat. Use this category when AV nodal beat origin is suspected: narrow complex QRS with a negative P-wave with a short PR intervals, no discernible P-wave, or a P-wave after the QRS. This beat type should be used both for junctional escape rhythms, premature junctional complexes, and junctional tachycardias.

- **V **– ventricular beat or series of beats, including premature ventricular complexes, escape rhythms, and competing ventricular rhythms. These will be sorted by the system into singles, couplets, ventricular tachycardias, IVR/AIVR runs, and bi/trigeminy episodes.

- **F** – fusion beat - Hybrid complexes from supraventricular and ventricular complexes should be marked as fusion beats regardless of QRS duration.
- **UNK** – unknown beat type. This annotation can be used when QRS complexes can be clearly discerned but the level of noise is such that the beat type can't be discerned. Try not to overuse. Short episodes of noise where the beats keep occurring at the expected rate and in the expected pattern can be classed as the rhythm type preceding the noise.

Second level labels

- **Aber** – aberrant conduction - this can be used in conjunction with any N, S, or AF beat, to describe aberrant conduction. We need these annotations in order to be able to handle broad complex tachycardias appropriately. Use this for clear cases of aberration, i.e. beats with a qualitative difference compared to the patients normal configuration or a clear bundle branch block configuration, and with a QRS duration >120ms.
- **Prex** – This should apply to beats of any supraventricular origin (N, S, or AF) that has evidence of pre-exitation, i.e. a delta wave. Mark only the beats with delta wave with Prex.
- **PM** – In case of pacing the first level classification should be the origin of the atrial impulse which is paced, for example N+PM for sinus rhythm P-waves with ventricular

pacing and AF + PM in paced AF. When pacing results in a paced P-wave which leads to a conducted QRS use N+PM. When pacing results in a paced P-wave and a wide QRS use N + PM + Aber. A rhythm with P-waves and paced ventricular/aberrant QRS complexes should be marked as N/S + PM + Aber. Ventricular pacing without any P-waves should be marked as V + PM. So:

  - paced, P-wave, narrow QRS -> N+PM
  - paced, P-wave, wide QRS -> N/S+PM+Aber
  - paced, no P-wave, wide QRS -> V+PM+Aber

- **2AVB1** – 2nd degree atrioventricular block Mobitz type I (Wenckebach phenomenon), i.e progressively lengthening PQ intervals until non-conduction of an impulse after which the pattern can repeat. Use the 2AVB1 annotation on top of beats with N as first level annotation, starting at the first prolonged PR interval, and mark all beats with 2nd degree AVB type 1 with the 2AVB1 label. Mark any escape beats (J or V) as such. Note: Very short Wenchebach phenomena with 2:1 conduction can't reliably be differentiated from AVB 2 type II with 2:1 conduction. In such cases, the AVB2 type II label should be used.
- **2AVB2** – 2nd degree atrioventricular block Mobitz type II. Use this label when the underlying rhythm is supraventricular in origin but there is non-conduction of some impulses, without a Wenchebach phenomenon. Use the AVB2 type 2 label on the QRS complex following the non-conducted p-wave, regardless of first level classification of that beat (N, J, or V), i.e., use the origin of the QRS complex to define the first level beat label, and add the 2AVB label as second level of the beat after the non-conducted impulse. Blocked premature ectopic P-waves should not be annotated as AVB.
- **3AVB** – 3rd degree atrioventricular block should be used for a rhythm with AV-dissociation, as a second level, where the first level is the origin of escape rhythm (J,V). Mark the whole episode with 3AVB, excluding normally conducted beats in case of intermittent 3rd degree AVB. For narrow complexes with 3AVB assume the QRS is of junctional origin and use J+3AVB. For broad complexes assume a ventricular origin and mark as V+3AVB.
- **SA** – sinus arrest. Use this second level annotation on any N, S, J or V beat following a ≥3 second pause due to sinus arrest (no P-wave) or a pause of any duration following a pattern indicative of sinoatrial exit block, with an appearance of either SAE block 2 type 1 (consecutively shorter P-P intervals until the loss of a P-wave), or SAE block 2 type 2, (intermittent loss of a P-wave with a pause surrounding the dropped P-wave corresponding to 2 P-P intervals). Don’t use this label following blocked P-waves.

Area Annotations

Area annotations are used for noise or a rhythm that does not result in discernible QRS complexes.

- **Unk** – unknown signal caused by: -- artifacts from patient movement, loose leads, or electromagnetic interference. -- disconnected electrodes - no signal. UNK area should be used when both ECG leads are completely unreadable. If noise is high, but you can still correctly guess annotation with 99% confidence then you can mark guessed beats. Otherwise mark unreadable fragment with “UNK beats” area, see example:

- VF – ventricular fibrillation. Not to be mistaken with VT – ventricular tachycardia, which must be marked with series of V beats. VF should be used for chaotic fibrillation where individual beats cannot be distinguished
- Blocked PAC –use this area in the case of blocked PACs to cover the blocked PAC. Please mark non- conducted P wave with area "blocked PAC" from beginning to the end of P wave ** Note **please don't use this area for 2AVB1 and 2AVB2 and for non-conducted PAC during AET.
