# Supplementary material for: Artificial intelligence for direct-to-physician reporting of ambulatory electrocardiography
Source: Nat Med. 2025 Feb 10;31(3):925–31. doi: 10.1038/s41591-025-03516-x (PMC11922735; doi:10.1038/s41591-025-03516-x)
Supplement: Supplementary file 2 — Reporting Summary [file 41591_2025_3516_MOESM2_ESM.pdf]

Corresponding author(s): Linda Johnson

Last updated by author(s): 25 November 2024

Reporting Summary

Nature Portfolio wishes to improve the reproducibility of the work that we publish. This form provides structure for consistency and transparency in reporting. For further information on Nature Portfolio policies, see our Editorial Policies and the Editorial Policy Checklist.

Statistics

For all statistical analyses, confirm that the following items are present in the figure legend, table legend, main text, or Methods section.

n/a	Confirmed	
☐	☒	The exact sample size (*n*) for each experimental group/condition, given as a discrete number and unit of measurement
☐	☒	A statement on whether measurements were taken from distinct samples or whether the same sample was measured repeatedly
☐	☒	The statistical test(s) used AND whether they are one- or two-sided<br>*Only common tests should be described solely by name; describe more complex techniques in the Methods section.*
☐	☒	A description of all covariates tested
☐	☒	A description of any assumptions or corrections, such as tests of normality and adjustment for multiple comparisons
☐	☒	A full description of the statistical parameters including central tendency (e.g. means) or other basic estimates (e.g. regression coefficient) AND variation (e.g. standard deviation) or associated estimates of uncertainty (e.g. confidence intervals)
☐	☒	For null hypothesis testing, the test statistic (e.g. *F*, *t*, *r*) with confidence intervals, effect sizes, degrees of freedom and *P* value noted<br>*Give P values as exact values whenever suitable.*
☒	☐	For Bayesian analysis, information on the choice of priors and Markov chain Monte Carlo settings
☒	☐	For hierarchical and complex designs, identification of the appropriate level for tests and full reporting of outcomes
☒	☐	Estimates of effect sizes (e.g. Cohen's *d*, Pearson's *r*), indicating how they were calculated

Our web collection on statistics for biologists contains articles on many of the points above.

Software and code

Policy information about availability of computer code

Data collection	Data extraction from ECGs was performed in Python v3.8.12, using custom written code and numpy 1.22.3, pandas 1.1.0, and torch 1.10.2 +cu113
Data analysis	Statistical analyses were mainly performed in Python v3.8.12, using custom written code and numpy 1.22.3, pandas 1.1.0, and torch 1.10.2 +cu113. Incidence rate ratios were calculated in Stat for Mac v 17.0 using epitab commands.

For manuscripts utilizing custom algorithms or software that are central to the research but not yet described in published literature, software must be made available to editors and reviewers. We strongly encourage code deposition in a community repository (e.g. GitHub). See the Nature Portfolio guidelines for submitting code & software for further information.

Data

Policy information about availability of data

All manuscripts must include a data availability statement. This statement should provide the following information, where applicable:

- Accession codes, unique identifiers, or web links for publicly available datasets
- A description of any restrictions on data availability
- For clinical datasets or third party data, please ensure that the statement adheres to our policy

The study data that support the findings of this study are not publicly available due to their potentially sensitive nature but will be made available from the corresponding author for the purpose of reviewing the study results, at the cost of a reasonable fee for data preparation. No requests that include a commercial

interest will be approved. Data are located in controlled access data storage at MEDICALgorithmics. A response to a request to access the data can be expected within two months.

Research involving human participants, their data, or biological material

Policy information about studies with human participants or human data. See also policy information about sex, gender (identity/presentation), and sexual orientation and race, ethnicity and racism.

Reporting on sex and gender	We report sub-analyses by sex for the most important study outcomes
Reporting on race, ethnicity, or other socially relevant groupings	We have no information regarding race, ethnicity or any other socially relevant grouping.
Population characteristics	The paper reports on an unselected patient population that has been referred to and undergone mobile cardiac telemetry in the US. The mean age is 65.5±10 years, and 42.8% of the population was male.
Recruitment	Patients were not specifically recruited for this study - they were included if they had undergone mobile cardiac telemetry in the US for a clinical indication, between 2016-2019, after anonymisation of the data.
Ethics oversight	The Ethics Review Board of Sweden has waived the need for an ethical approval, considering the anonymised nature of the data, decision number 2019-03227

Note that full information on the approval of the study protocol must also be provided in the manuscript.

Field-specific reporting

Please select the one below that is the best fit for your research. If you are not sure, read the appropriate sections before making your selection.

☒ Life sciences ☐ Behavioural & social sciences ☐ Ecological, evolutionary & environmental sciences

For a reference copy of the document with all sections, see nature.com/documents/nr-reporting-summary-flat.pdf

Life sciences study design

All studies must disclose on these points even when the disclosure is negative.

Sample size	We used the maximal population size of unselected patient recordings never before seen by the algorithm that was available to us (n=14,606 patient recordings). The number of strips annotated was pre-determined based on three criteria: 1. The maximal sample size that we could annotate using gold standard methodology, assuming that each annotator would spend a maximum of 40 hours to annotate the data, and the number of physicians in our network that were willing to contribute to the project. 2. What we considered to be a reasonable number of arrhythmic events in order to capture a diverse sample of electrographic presentations (minimum 250, but with a larger sample for critical arrhythmia events). 3. The maximum number of arrhythmic events found in the population sample of 14,606 patients that was available to you.
Data exclusions	No data was excluded
Replication	No external dataset was available to us for replication
Randomization	Arrhythmic episodes included in the study were randomly selected within each individual. No randomization was performed at the patient level - all ECG monitoring sessions were analysed with both DeepRhythmAI and ECG technician analysis (usual care)
Blinding	The annotators were blinded to the methods by which the strips included in the study were selected (except LSJ, A Benz, and JH who designed the study) and all annotators were blinded to whether they were annotating an AI or technician selected strip as well as to what the AI or technician annotations were.

Reporting for specific materials, systems and methods

We require information from authors about some types of materials, experimental systems and methods used in many studies. Here, indicate whether each material, system or method listed is relevant to your study. If you are not sure if a list item applies to your research, read the appropriate section before selecting a response.

Materials & experimental systems

n/a	Involved in the study
☒	☐ Antibodies
☒	☐ Eukaryotic cell lines
☒	☐ Palaeontology and archaeology
☒	☐ Animals and other organisms
☒	☐ Clinical data
☒	☐ Dual use research of concern
☒	☐ Plants

Methods

n/a	Involved in the study
☒	☐ ChIP-seq
☒	☐ Flow cytometry
☒	☐ MRI-based neuroimaging

Plants

Seed stocks

Report on the source of all seed stocks or other plant material used. If applicable, state the seed stock centre and catalogue number. If plant specimens were collected from the field, describe the collection location, date and sampling procedures.

Novel plant genotypes

Describe the methods by which all novel plant genotypes were produced. This includes those generated by transgenic approaches, gene editing, chemical/radiation-based mutagenesis and hybridization. For transgenic lines, describe the transformation method, the number of independent lines analyzed and the generation upon which experiments were performed. For gene-edited lines, describe the editor used, the endogenous sequence targeted for editing, the targeting guide RNA sequence (if applicable) and how the editor was applied.

Authentication

Describe any authentication procedures for each seed stock used or novel genotype generated. Describe any experiments used to assess the effect of a mutation and, where applicable, how potential secondary effects (e.g. second site T-DNA insertions, mosiacism, off-target gene editing) were examined.
